# Tumor absorbed dose and outcomes after Y-90 glass radioembolization for colorectal liver metastases: does dose escalation improve survival in real-world practice?

**DOI:** 10.1007/s00259-026-07933-z

**Published:** 2026-05-22

**Authors:** Osman Melih Topcuoglu, Betul Uzunoglu, Tolga Orhan, Ismail Necim Yucel, Ayşegul Gormez, Turkay Toklu, Nalan Alan Selcuk

**Affiliations:** 1 Present Address: Department of Radiology, Yeditepe University Hospitals, Kosuyolu, Istanbul, 34718 Turkey; 2 Department of Nuclear Medicine, Yeditepe University Hospitals, Kosuyolu, Istanbul, 34752 Turkey

**Keywords:** Colon cancer, Liver metastasis, Radioembolization, Yttrium-90

## Abstract

**Purpose:**

To assess the dose-response and dose-survival outcomes of yttrium-90 (Y90) transarterial radioembolization (TARE) using Therasphere microspheres in patients with colorectal cancer liver metastases (CRCLM).

**Material and methods:**

Between May 2014 and December 2024, patients with chemorefractory CRCLM who underwent Y90 TARE using glass microspheres were retrospectively included. Patients were divided into two groups according to tumour absorbed dose (TAD): TAD<189 Gy (group 1) and TAD ≥189 Gy (group 2). The primary outcomes were overall survival (OS) and hepatic progression-free survival (hPFS). The secondary outcome was the objective response rate (ORR).

**Results:**

A total of seventy-eight patients with a mean age of 69.9±11.2 years (range 41 to 91 years) were included. The median tumor dose and the median OS were 171.5 Gy (IQR; 147-200 Gy) and 8.9 months ( IQR; 5-14 months), respectively. The median TAD for groups 1 (n=46) and 2 (n=32) was 150 Gy (IQR; 122-166 Gy) and 202 Gy (IQR; 195-232 Gy), respectively. The median OS and hPFS for group 1 versus group 2 were 9.4 months (IQR; 6.7–17.7 months) and 5.2 months (IQR; 4-6.7 months) versus 7.1 months (IQR; 5–10.7 months) and 5.5 months (IQR; 4.2-6.7 months), respectively and were not significantly different (p = .107 and p = .663, respectively). The ORR did not differ significantly between the two groups (p = .943).

**Conclusion:**

In a real-world population of heavily pretreated patients with CRCLM, escalation of TAD beyond 189 Gy did not translate into improved survival, highlighting the dominant impact of advanced disease burden and extrahepatic progression.

## Introduction

Colorectal cancer (CRC) remains the fourth most common type of cancer. Despite the recent development of new drug technologies, it remains one of the leading causes of cancer-related death [1]. Between 50% and 70% of cases develop liver metastases during the course of the disease, significantly impacting survival and quality of life [2]. For patients with unresectable colorectal cancer liver metastases (CRCLM), systemic chemotherapy remains the mainstay of treatment. However, outcomes are often limited in the chemorefractory setting, highlighting the need for effective liver-directed therapies [3].

Yttrium-90 (Y-90) transarterial radioembolization (TARE) has emerged as a valuable therapeutic option for patients with liver-dominant, chemotherapy-resistant CRCLM. By delivering high-dose beta radiation directly to hepatic tumors while sparing non-tumorous parenchyma, TARE offers the potential for local tumor control with an acceptable safety profile [4]. Previous studies have suggested that the efficacy of treatment may be influenced by the tumor absorbed dose (TAD), with higher radiation doses hypothesised to improve both the objective tumor response and survival outcomes [5–7]. Furthermore, Alsultan et al. [5] stated that CRCLM receiving a TAD of exceeding 189 Gy were shown to have a complete response. Soydal et al. [7] reported that patients receiving a TAD of more than 203 Gy had better overall survival (OS) compared to those receiving less than 203 Gy. We hypothesized that patients receiving TAD ≥ 189 Gy would demonstrate improved OS, hPFS, and ORR compared to those receiving lower doses, based on prior threshold-based studies. However, the aim of this study was not to establish a novel tumor absorbed dose threshold, but to evaluate the real-world applicability of a guideline-referenced TAD threshold in a heterogeneous, heavily pretreated population with CRCLM. The purpose of the current study was to assess the dose-response and dose-survival outcomes of Y-90 TARE using Therasphere microspheres in patients with CRCLM.

## Materials and methods

### Patient population

**Fig. 1:**
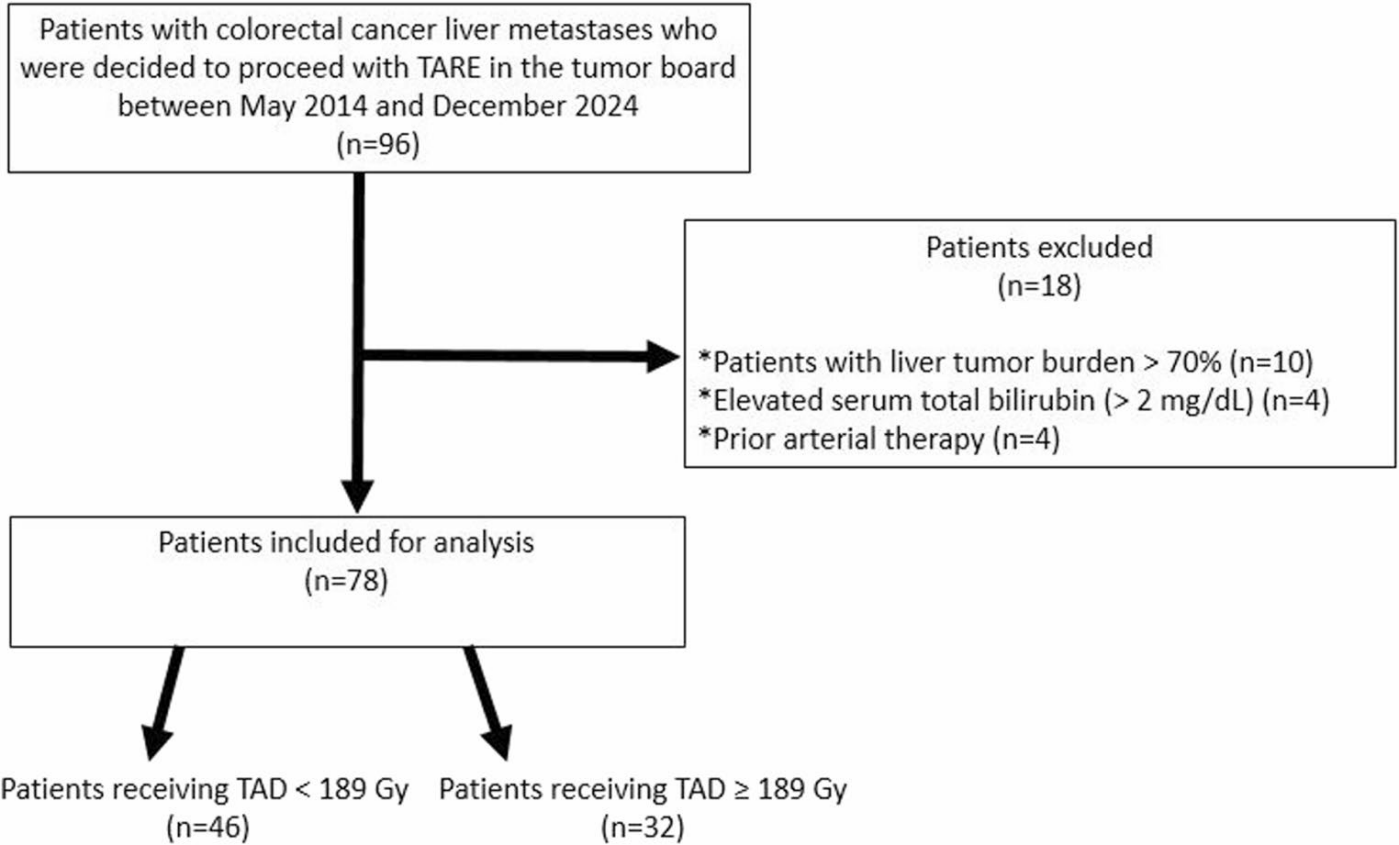
The study flow chart. *TARE; transarterial radioembolization*,* TAD; tumor absorbed dose*

The local Institutional Review Board (IRB) approved this single center study and the need for patient consent was waived. Between May 2014 and December 2024 patients with chemo-refractory hepatic metastases from colorectal cancer who underwent Y-90 TARE with glass microspheres, were retrospectively included. All patients had progressive disease following first- and second-line chemotherapy. The following criteria excluded subjects from the study: Patients with tumor burden greater than 70% of the liver, a life expectancy of 3 months or less, an Eastern Cooperative Oncology Group (ECOG) performance status of > 2, elevated total serum bilirubin (> 2 mg/dL) and decreased serum albumin (< 2.5 g/dL).Patients who had received prior liver-directed therapies such as chemoembolization and radioembolization treatments, were also excluded (Fig. 1).

### TARE procedure

A multidisciplinary tumour board made the decision regarding each patient’s treatment. Antibiotic prophylaxis was not used. The TARE procedures were performed by an interventional radiologist who had over nine years’ experience of Y90 procedures. Planning angiograms using 99mTc-macroaggregated albumin (99mTc-MAA) were performed 7–10 days prior to TARE. The intra- and extrahepatic distribution of 99mTc-MAA was evaluated using single-photon emission computed tomography (SPECT/CT). The decision to proceed with TARE was made if the lung shunt fraction (LSF) and the expected lung doses were within the accepted limits, and if the tumors exhibited greater MAA avidity than the normal liver parenchyma. The tumor-to-normal parenchyma ratio was calculated in order to determine the doses of tumor and normal tissue using the partition model with the commercially available Simplicit90Y^®^ software (Mirada Medical Ltd. Oxford, UK). All procedures were performed via the standard transfemoral route under local anesthesia. A 5 F catheter (USL-2; Uni Select 2 SLX™, Cordis, Miami Lakes, Florida, USA) was used to catheterize the celiac trunk. A 2.4–2.7 F microcatheter (Progreat, Terumo, Somerset, New Jersey, USA) was used for the selective catheterization of the index hepatic artery. Y-90- containing microspheres (TheraSphere, Y90 microspheres, Boston Scientific, Marlborough, MA) were delivered from the same catheter site. Lobar or sequential bi-lobar treatment was performed. Cone-beam CT was used for every patient. Both 1st and 2nd week Therasphere vials with baseline activities ranging from 3 to 15 GBq at calibration (corresponding to ~ 4,000 Bq/sphere at calibration) were ordered depending on treatment scheduling, reflecting routine clinical practice. Week 2 vials contain a higher number of microspheres per administered activity, which may influence embolic behavior and intrahepatic distribution. Post-90Y positron emission CT (PET/CT) scans were obtained after each treatment, and post-procedure dosimetry was calculated. For patients undergoing sequential bi-lobar treatment, separate 99mTc-MAA simulation procedures were performed before each treatment session. During each session, the microcatheter was positioned at the intended treatment site and MAA was administered to reflect the perfusion territory of the relevant hepatic lobe. This enabled dosimetric planning specific to each lobe, accounting for potential differences in arterial flow and changes in hepatic hemodynamics over time.

### Dose calculation

TAD was calculated from the 99mTc-MAA SPECT-CT images using a partition model with respect to the medical internal radiation dose (MIRD) approach. The target volume and LSF obtained from the Tc-MAA SPECT/CT study were used to calculate the TAD. Tumor delineation and image evaluation were performed using Simplicit90Y^®^ software (Mirada Medical Ltd. Oxford, UK). Tumor segmentation was performed on a lesion-by-lesion basis using co-registered SPECT/CT images. Individual lesion volumes of interest were manually delineated with consensus between experienced readers. TAD was initially calculated for each lesion separately based on activity concentration and lesion volume. For the patient-level analysis, the median TAD value was calculated for each patient based on all measurable lesions. This value was used for grouping (< 189 Gy vs. ≥ 189 Gy) and for survival analyses. In the latter, the median TAD per patient was employed as a summary metric, whereas individual lesion TAD values were retained for dosimetric calculations. Post-treatment dosimetry was performed using Y-90 PET/CT acquired after radioembolization. Quantitative analysis was conducted using the same software platform to ensure methodological consistency between pre- and post-treatment assessments. In sequential bi-lobar treatments, TAD calculations were based on the corresponding lobe-specific MAA simulation for each treatment session.

### Definitions of outcomes

Imaging was performed using fluorodeoxy glucose (FDG) PET at baseline and every 3 months thereafter. The response outcome was assessed after 3 months. The objective response rate (ORR) (complete metabolic response; CMR, partial metabolic response; PMR), stable disease; SD and progressive metabolic disease; PMD) were determined according to the PET response criteria in solid tumors (PERCIST) [8]. CMR means complete resolution of FDG uptake in the target lesions. PMR means ≥ 30% reduction in the maximum standard uptake value (SUVmax), PMD means ≥ 30% increase in the SUVmax or the appearance of new FDG-positive lesions that are typical for malignancy in the treated lobe and SD means that the changes that do not meet the criteria for CMR, PMR, or PMD. The baseline and post-treatment images were evaluated by two radiologists (one with three years’ experience and one with more than 10 years’ experience in liver imaging) and a nuclear medicine specialist with 25 years’ experience in PET imaging. The evaluation was conducted by consensus. A response to Y-90 TARE was defined as CMR or PMR, whereas a non-response was defined as SD or PMD. The disease control rate (DCR) was defined as CMR + PMR+SD. hPFS was defined as the time interval from TARE to radiologic evidence of disease progression within the liver or death, whichever occurred first. Hepatic progression was assessed on follow-up FDG PET/CT examinations performed at 3-month intervals and was defined according to PERCIST criteria as PMD within the treated liver, including an increase in SUVmax ≥ 30% in existing hepatic lesions or the appearance of new FDG-avid hepatic lesions. Extrahepatic progression alone was not considered an event for hPFS. Patients were divided into two groups: group 1 received a TAD of less than 189 Gy, and group 2 received a TAD of 189 Gy or more. The Common Terminology Criteria for Adverse Events (CTCAE v5.0) was used to define clinical and laboratory adverse events.

### Statistical analysis

Categorical variables were expressed as numbers (percentages) and continuous variables as medians (interquartile ranges; IQR). In addition to threshold-based comparisons, TAD was also analyzed as a continuous variable in relation to OS and hPFS using Cox regression models. A Shapiro-Wilk normality test was performed on the continuous variables. A chi-squared test and a Mann–Whitney U-test were used to assess the differences between the groups. The confidence interval was set at 95%. Kaplan-Meier analysis was used to assess OS and hPFS. The log-rank test was used to compare OS and hPFS between groups. Hazard ratios (HRs) with 95% confidence intervals (CIs) for OS and hPFS were calculated using Cox regression. OS was defined as the time between TARE and death from any cause. A P-value of < 0.05 was considered significant. Pearson correlation and linear regression analysis were used to evaluate the correlation between pre-treatment and post-Y90 dosimetry, with the regression slope and intercept reported to assess potential systematic bias between the two methods. All analyses were performed using SPSS 25.0 (IBM, Statistical Package for the Social Sciences, Armonk, N.Y., USA). Dose–outcome analyses were conducted at the patient level for patients with multifocal disease. These analyses used the median TAD and did not account for individual lesion volume or underdosed lesions. Although this approach reflects real-world clinical decision-making, it does not account for heterogeneity in doses received by different patients.

## Results

**Fig. 2:**
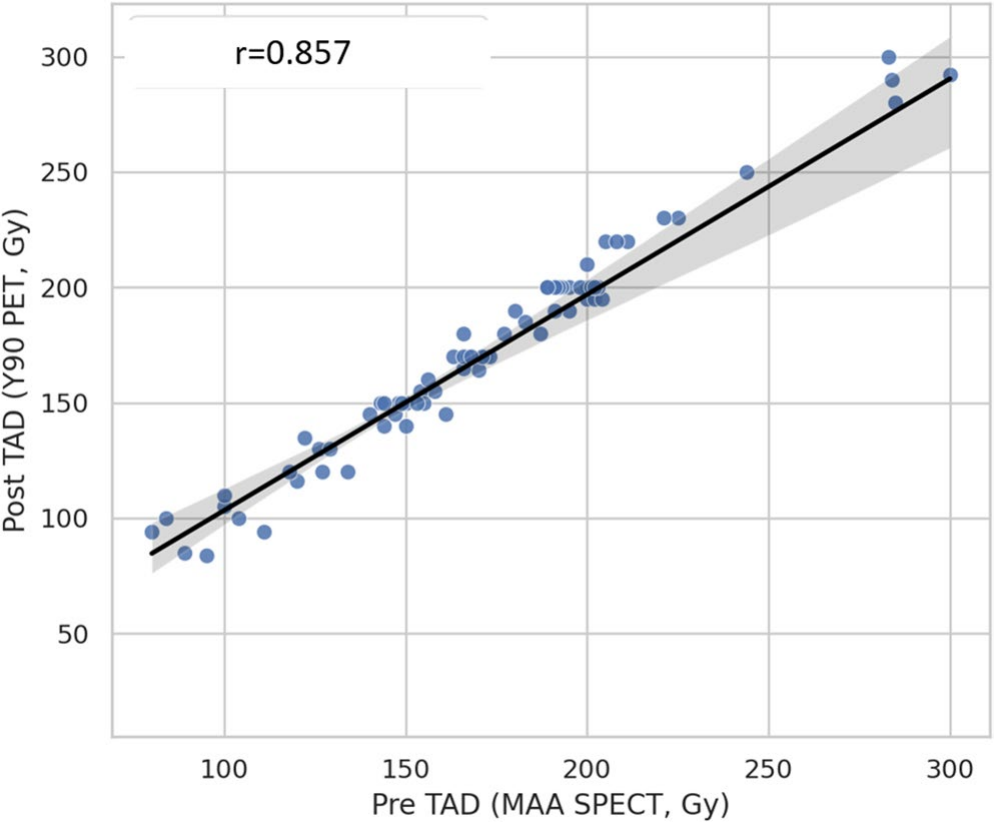
Graphic shows the slope and intercept of the linear regression between MAA-based and PET-based TAD. *MAA; macro-albumin aggregate*,* PET; positron emission tomography*,* TAD; tumor absorbed dose*

Out of 96 patients, 78 met the inclusion criteria, with a mean age of 69.9 ± 11.2 years (range 41–91 years). Of these patients, 73.1% (57/78) had extrahepatic disease and 85.9% (67/78) had bilobar disease. Most patients had liver involvement of at least 50%, and this was the case for over half of the cohort. There were no differences between groups with regard to these variables (Table 1). The Shapiro–Wilk normality test showed *p* < .05. The median tumor dose was 171.5 Gy (IQR; 147–200 Gy). The median background liver doses were 64.7 Gy and 77 Gy for groups 1 and 2, respectively (*p*= .095). The median TAD for groups 1 (*n* = 46) and 2 (*n* = 32) was 150 Gy (IQR; 122–166 Gy) and 202 Gy (IQR; 195–232 Gy), respectively (*P*<.001). A total of 48 patients underwent lobar treatment, while 30 patients underwent sequential bi-lobar treatment. 65 (83.3%) of patients had received prior bevacizumab therapy as part of prior systemic treatment regimens, in line with standard treatment protocols for metastatic colorectal cancer. Of the 78 patients, 51 (65.4%) received week 1 vials and 27 (34.6%) received week 2 vials. Post-Y90 dosimetry showed a good correlation with the pre-treatment dosimetry values (r: 0.857) (Fig. 2). Linear regression analysis revealed a strong correlation between MAA- and PET-based TAD estimates (*r* = .857). The regression slope was found to be 0.934, and the intercept was found to be 9.940. These results suggest that there is no significant systematic bias between the two methods. When comparing pre-treatment MAA-based dosimetry with post-treatment Y-90 PET-based dosimetry, no systematic bias leading to reclassification across the predefined 189 Gy threshold was observed. Using post-therapy dosimetry did not result in patients being reclassified across the 189 Gy threshold.

**Table 1:** Characteristics of the study cohort

Characteristic	Group 1 (*n* = 46)	Group 2 (*n* = 32)	*P* value
Median age, years (IQR)	71 (60–80)	72 (64–76)	0.763
ECOG Performance status, n (%)			0.303
0	40 (86.9)	25 (78.1)	
1	6 (13.1)	7 (32.9)	
Tumor Distribution, n (%)			0.325
Unilobar	5 (1.9)	6 (18.7)	
Bilobar	41 (89.1)	26 (81.3)	
Extrahepatic metastases, n (%)			0.841
Yes	34 (73.69)	23 (71.9)	
No	12 (26.1)	9 (28.1)	
KRAS mutation n (%)			0.587
Yes	12 (26.1)	6 (18.7)	
No	34 (73.9)	26 (81.3)	
Primary tumor side, n (%)			0.431
Right colon	10 (21.7)	10 (31.3)	
Left colon	36 (78.3)	22 (68.7)	
Y90-Tx Side n (%)			0.276
Right	30 (65.2)	20 (62.5)	
Left	16 (34.8)	12 (37.5)	
Liver tumor volume (%)			0.411
<30%	9 (19.5)	10 (31.2)	
30% ≤ and < 50%	9 (19.5)	7 (21.9)	
50% ≤ and < 70%	28 (61)	15 (46.9)	

*Gy* gray, *Tx* treatment

### Survival outcomes

**Fig. 3:**
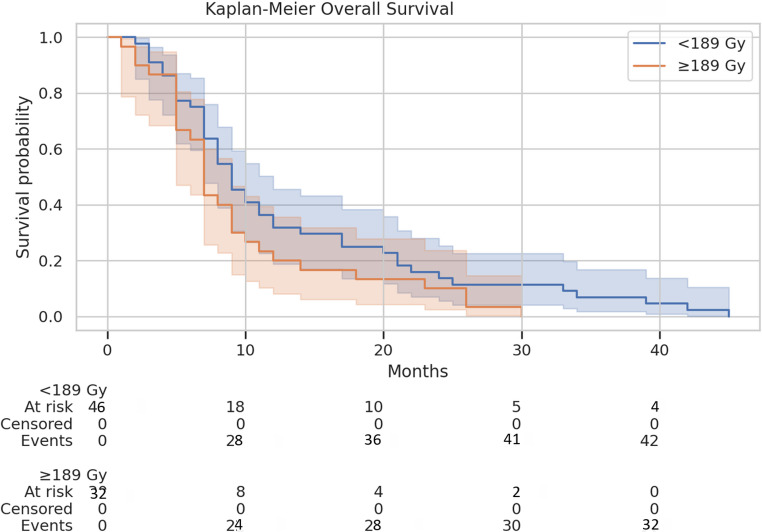
Kaplan-Meier analysis of overall survival for patients receiving TAD ≥ 189 Gy versus TAD < 189 Gy. *TAD; tumor absorbed dose*

**Fig. 4:**
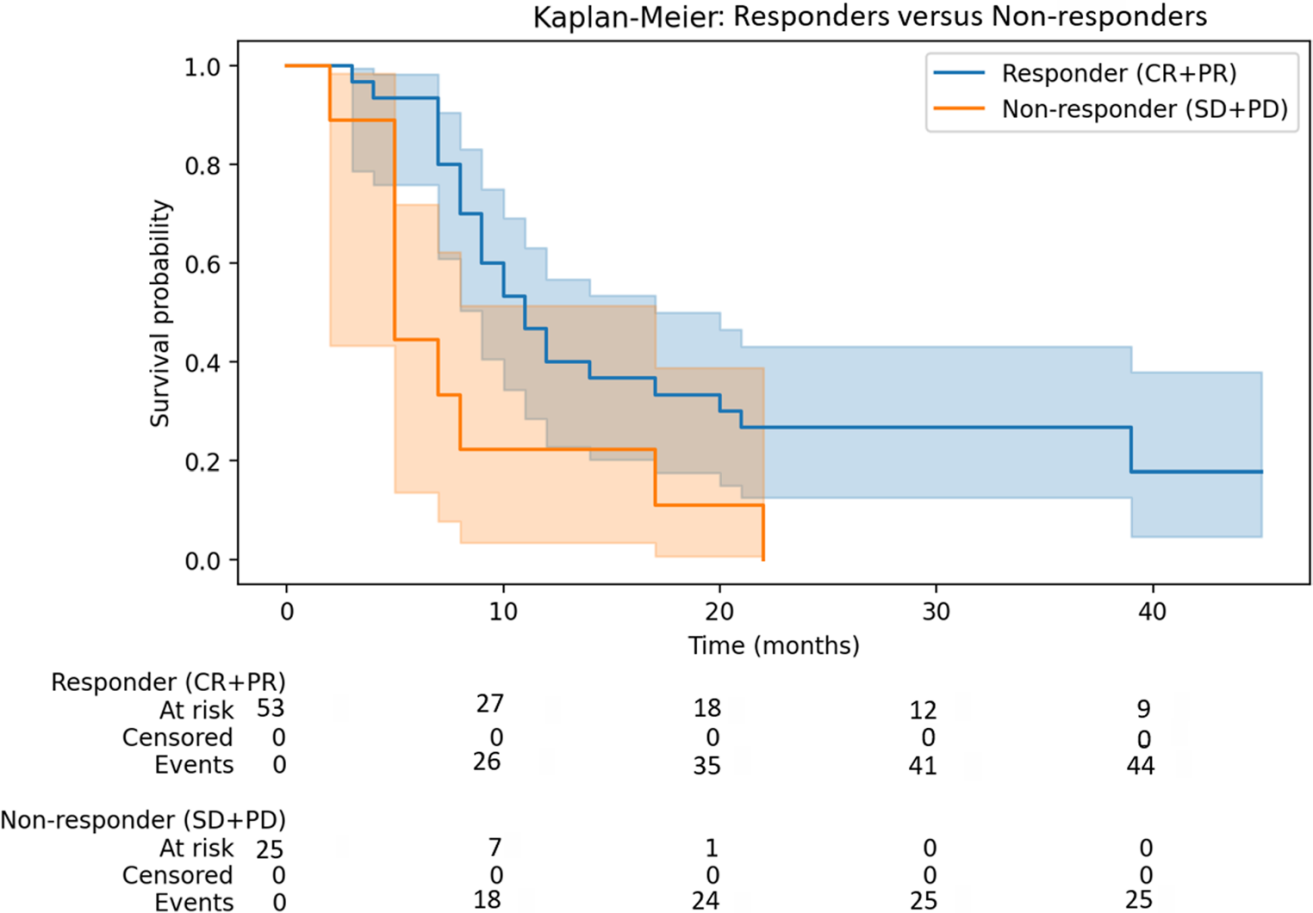
Kaplan-Meier analysis of overall survival for responders and non-responders

**Fig. 5:**
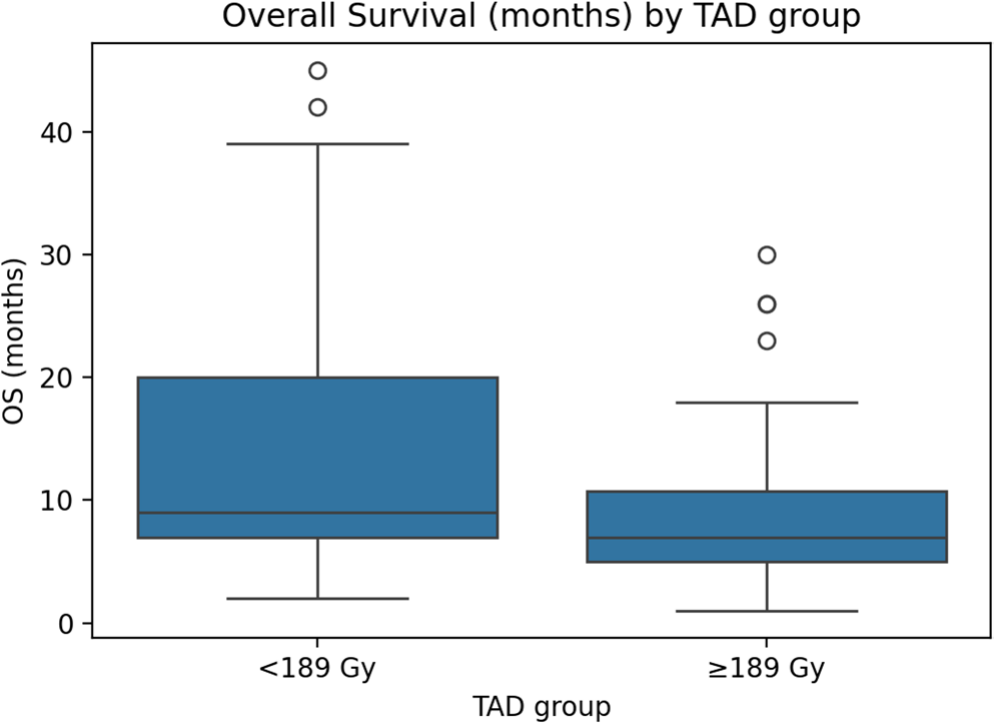
The plot diagram shows the relationship between tumor absorbed dose and overall survival. *OS; overall survival*,* TAD; tumor absorbed dose*

**Fig. 6:**
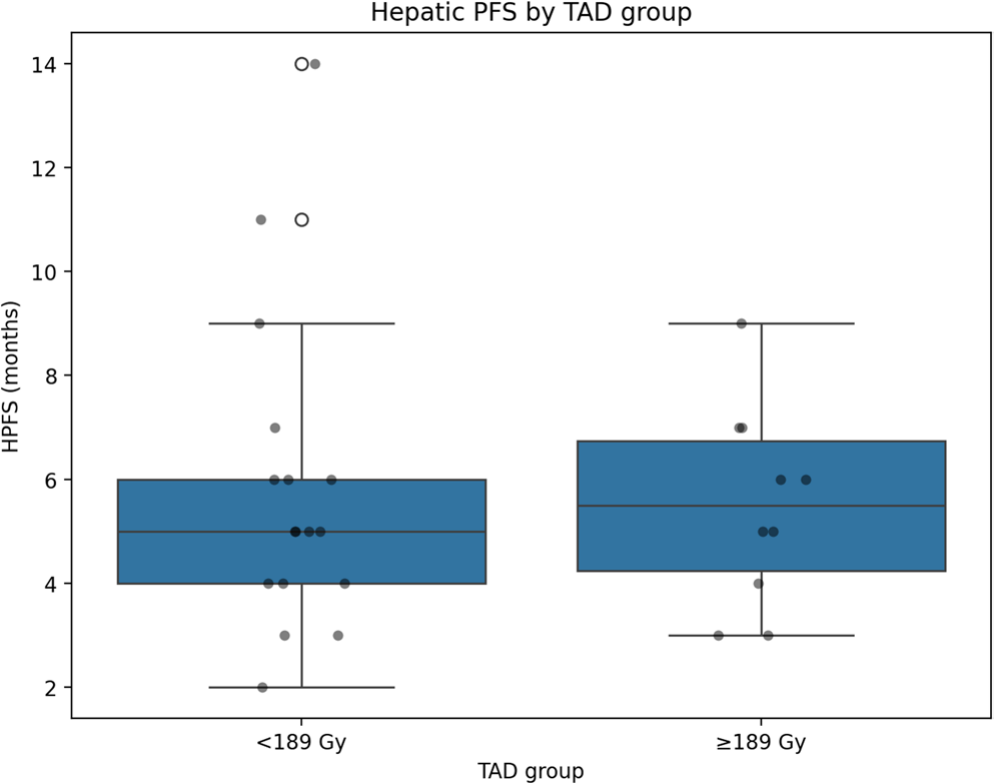
The plot diagram shows the relationship between tumor absorbed dose and hepatic progression-free survival. *hPFS; hepatic progression free survival*,* TAD; tumor absorbed dose*

**Fig. 7:**
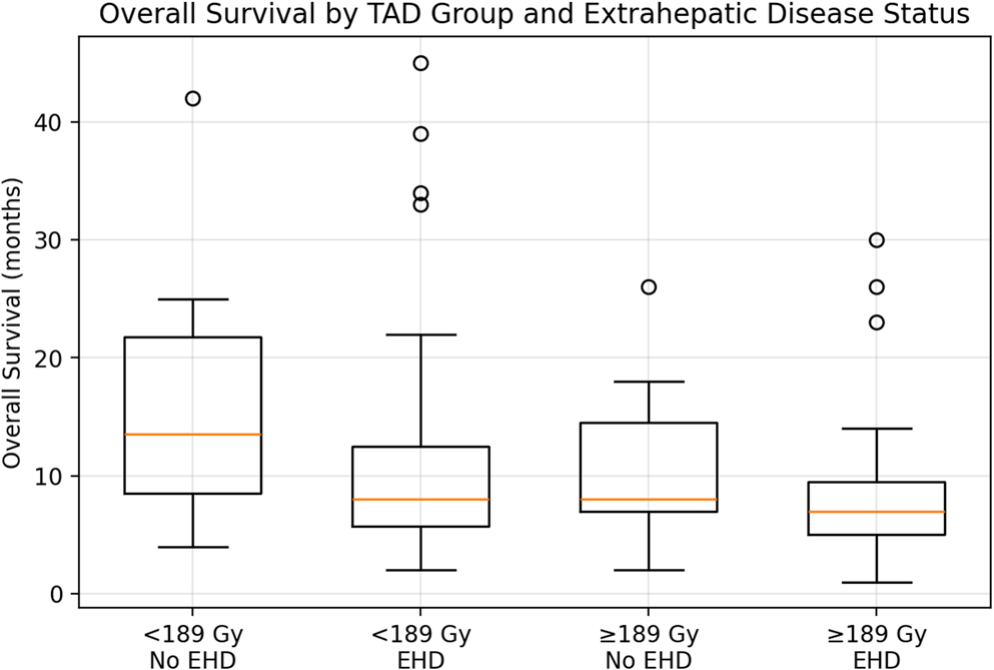
The plot diagram shows the relationship between overall survival, tumor absorbed dose and the presence of extrahepatic disease. *OS; overall survival*,* TAD; tumor absorbed dose*,* EHD; extrahepatic disease*

**Fig. 8:**
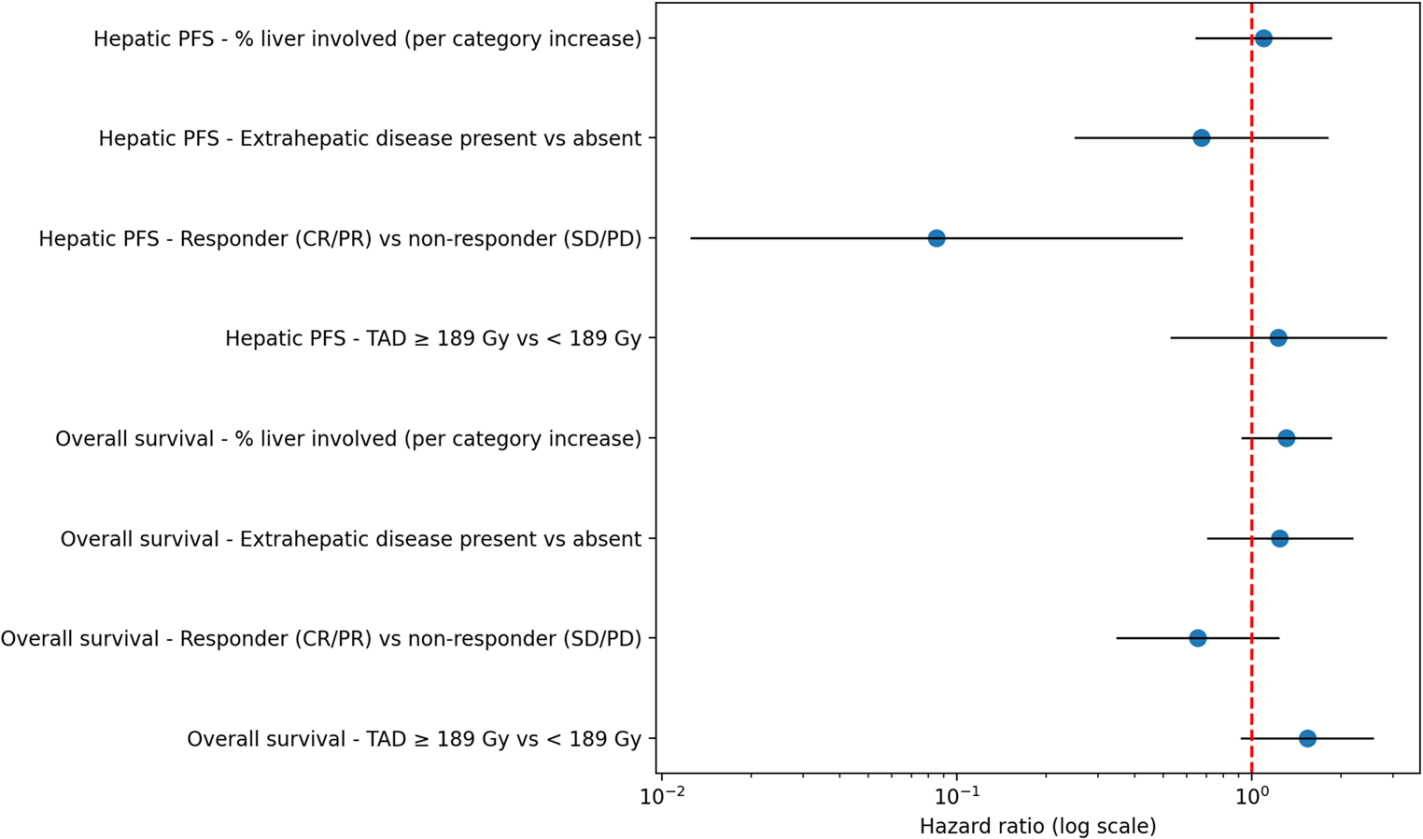
The forest graphic shows each variable’s hazard ratio (point) with its 95% confidence interval (horizontal line), on a log scale, for both overall survival and hepatic progression free survival (PFS)

The median OS and hPFS for the whole cohort were 8.9 months (IQR; 6.75–17.75 months) and 5.1 months (IQR; 4.0–6.75 months), respectively. The median OS and hPFS for group 1 versus group 2 were 9.4 months (IQR; 6.7–17.7 months) and 5.2 months (IQR; 4-6.7 months) versus 7.1 months (IQR; 5–10.7 months) and 5.5 months (IQR; 4.2–6.7 months), respectively and were not significantly different (*P* = .107 and *P* = .663, respectively). The median OS in patients with and without extrahepatic disease was 8.6 months (IQR; 7–21 months) and 10.3 months (IQR; 7–10 months), respectively (*p* = .114). The median OS and hPFS for responders versus non-responders were 9.1 months (IQR; 7–17 months), and 5.5 months (IQR; 4.3–7 months), versus 7.3 months (IQR; 5–11.8 months), and 3 months (IQR; 3–3 months), respectively (*p* = .141 and *p* = .052, respectively). For OS, the HR for the TAD group (≥ 189 Gy vs. < 189 Gy), was 1.49 (95% CI 0.93–2.41, *p* = .104). For hPFS, the HR for progression in the ≥ 189 Gy vs. < 189 Gy was 1.22 (95% CI 0.55–2.71, *p* = .632). Kaplan-Meier analyses and box-plots were shown in Figs. 3, 4, 5, 6, 7 and 8.

### Objective response rate outcomes

**Fig. 9:**
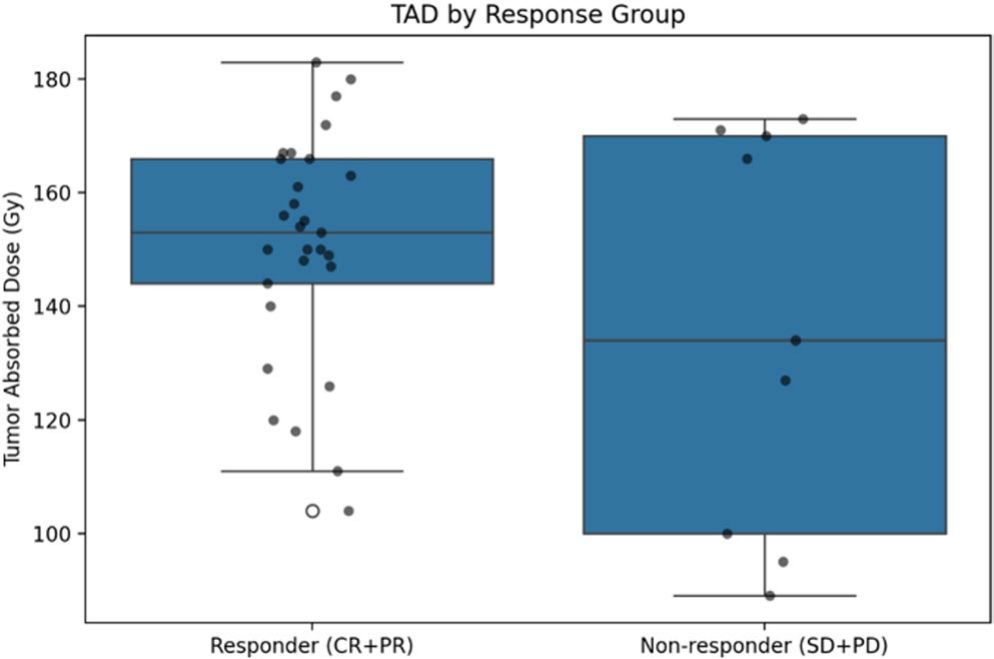
Schematic diagram shows TAD distribution for responders versus non-responders

The median follow-up period for all patients was 8.4 months (IQR; 5–14.8 months). The ORR and DCR for the entire cohort were 67.9% and 70.5%, respectively. There was no significant difference in the ORR between the two groups (*P* = .943). Of the patients, 53 (67.9%) were responders (CMR: *n* = 23; PMR: *n* = 30), and 25 (32.1%) were non-responders (PMD: *n* = 16; SD: *n* = 9). The median TAD for the responders and non-responders was 174.5 Gy (IQR; 150.8–200 Gy) and 172 Gy (IQR; 132,2–200 Gy), respectively (*p* = .632) (Fig. 9).

### Adverse events

Adverse events of grades 1, 2 and 3 were observed in 68 (87.2%), 26 (33.3%) and 8 (10.2%) patients, respectively. Two patients receiving TAD of 205 Gy (background liver dose of 55.3 Gy) and 166 Gy (background liver dose of 68.7 Gy) developed grade 3 adverse events in the form of ascites. An increase in γ-glutamyltransferase of more than ten times the baseline value was observed in six patients experiencing a grade 3 adverse event. Grade 1 and 2 adverse events included laboratory toxicities (increases in aspartate aminotransferase, alanine transaminase increase, alkaline phosphatase and γ-glutamyltransferase increase) and clinical toxicities (fatigue, nausea and abdominal pain). The results are summarized in Table 2.

**Table 2:** The summary of the study results

Parameter	Group 1 (*n* = 46)	Group 2 (*n* = 32)	*P* value
TAD (Gy, median; IQR)	150 (122–166)	202 (195–232)	< 0.001
Background liver dose (Gy, median; IQR)	64.7 (52.0-77.8)	77 (55.6–95.8)	0.095
OS (months, median; IQR)	9.4 (6.7–17.7)	7.1 (3.1–6.3)	0.107
hPFS (months, median; IQR)	5.2 (4-6.7)	5.5 ± (4.2–6.7)	0.663
Grade 3 AE n (%)	5 (10.8)	3 (9.4)	0.574

*TAD* tumor absorbed dose, *Gy* gray, *OS* overall survival, *hPFS* hepatic progression free survival, *ORR* objective response rate, *AE* adverse event

## Discussion

The current study demonstrated that OS and hPFS were not significantly different between patients receiving TAD < 189 Gy and those receiving TAD ≥ 189 Gy in a real-world population. Alsultan et al. [5] found an improved but non-significant OS difference for patients receiving more than 189 Gy compared to those receiving less than 189 Gy. They also stated that a threshold of TAD over 189 Gy was found for a complete metabolic response to TARE in 24 patients with CRCLM. However, the median TAD did not differ significantly between responders and non-responders in the present study. The discrepancy may be due to several factors. Firstly, the small sample size of their study could be the main reason. Secondly, the dosimetry methods differed: a multicompartment method was used in the current study whereas a 1-compartment model was used in the previous study. Finally, the extrahepatic disease burden was higher in the present cohort (73.1%) than in the previous study (39%), which could have a negative impact on OS. In a multicenter retrospective study. Soydal et al. [7] found that a threshold of 152 Gy predicted a longer OS in patients with CRCLM who underwent glass particle-TARE. In contrast to their study, however, the patients included in the current study had more extrahepatic disease. As in the present study, OS did not differ significantly between responders and non-responders. Although TAD and TAD thresholds may play a key role in predicting OS in CRCLM, OS itself is actually not directly related to the absorbed dose threshold. In fact the threshold predicts the response, which is then indirectly related to survival. The results of the current study suggest that, in routine clinical practice, the survival benefit of escalating TAD beyond a certain threshold may be limited and potentially influenced by multiple confounding clinical and biological factors. Accordingly, the absence of a survival benefit with dose escalation should be interpreted in the context of validating a guideline-referenced threshold in real-world practice rather than defining a new biological dose–response cutoff.

The EPOCH phase III trial demonstrated that the implementation of TARE in second-line chemotherapy improved ORR and hPFS, but did not improve OS [9]. Emmons et al. [10] emphasized that TARE enhances OS in patients with liver-dominant metastatic colorectal cancer when employed as a second-line therapy in the RESIN registry. They found that the median OS for the entire cohort was 15 months. When TARE was used as a second-line treatment, this increased to 17.4 months. When TARE was used as a third-line therapy, as in the current study, they found a median OS of 12.5 months. Compared to the median OS of eight months in the present study, the increased OS was due to the their study cohort including only 38% of patients with extrahepatic disease and 72% having a tumour burden in the liver of less than 25% [10]. On the other hand, 73% of patients in the current study had extrahepatic disease and 24.4% of patients had a tumour burden of less than 30%, which is in line with real-world practice. Furthermore, although the resin microspheres were used in their study, the TAD values were lacking. In contrast to real-world practice, randomized clinical trials and multicentre registries enrol ideal patients with low or no extrahepatic disease and a diminished percentage of liver tumour burden. Therefore, the results of these studies may not reflect those of patients in routine clinical practice [11].

Van den Hoven et al. [12] found a significant dose-response relation in patients with CRCLM who were treated with resin microspheres. However, the TAD values were quite low at the time of the study ranging from 7 Gy to 174 Gy and response was detected in 37% of the patients. Doyle et al. [13] stated that the TAD was the most significant predictor of ORR, particularly noting that a dose of 120 Gy using resin microspheres was estimated to achieve an ORR of 55% at the 6-month follow-up. However, they only evaluated one or two index lesions, rather than whole metastases. However, despite these findings, the present cohort did not demonstrate a measurable survival advantage for patients who received a TAD of at least 189 Gy. There are several reasons that may explain this discrepancy. These include differences in patient selection (the current cohort contained a very high proportion of patients with extrahepatic disease and bilobar disease), the timing of TARE within the treatment course (many dose–response publications examined earlier or intermediate-line treatments, disease that was more limited to the liver), and differences in dosimetry metrics and microsphere types (glass versus resin) that alter microdosimetry [9–11]. The data from this study showed a similar median TAD for responders and non-responders (174.5 Gy vs. 172 Gy), which supports the hypothesis of an early plateau in response probability for many lesions. It should be noted at this point that, as these studies used resin microspheres, the dose thresholds must be interpreted in the context of the specific device and dosimetric methodology employed.

OS in chemorefractory CRCLM is heavily influenced by systemic progression and performance status. In the current cohort, 73.1% of patients had extrahepatic disease and 55.1% had a liver tumor burden of more than 50% of the total liver volume. Therefore, the potential survival benefit of superior intrahepatic control is likely to be reduced. Several prior works that reported OS benefits with higher TAD included populations with more liver-dominant tumours or a lower tumour burden, or involved earlier-line interventions, where intrahepatic disease had a greater influence on survival [9–15]. This reconciles the negative OS findings of the present study with literature showing improved local control but an inconsistent impact on OS when systemic disease is uncontrolled. Dimepoulos et al. [14] also emphasized that an increased number of the hepatic metastases had a detrimental effect on OS. Dabrowiecki et al. [15] investigated the timing of Y90 TARE in patients with CRCLM who experienced disease progression following first-line chemotherapy. They found that, when implemented as a second-line therapy, Y90 TARE resulted in improved OS, as well as a lower hepatic tumour burden, an absence of genetic mutation, and left-sided primary tumors. A low tumour metastasis burden has been shown to be associated with better local control and, eventually, prolonged OS [16]. A wide variety of factors including tumor differentiation, extrahepatic disease, tumor volume in the liver, albumin and bilirubin levels, performance status of the patient, prior treatments, and tumor biomarkers can affect OS in patients with CRCLM who have been previously heavily treated before TARE [17].

The observed toxicity rates (grade ≥ 3: 10.2%) in the current cohort were consistent with the previously reported safety profiles of glass microspheres [7, 10]. The available literature suggests that the background liver dose (the non-tumoral liver absorbed dose) is a stronger predictor of clinically relevant hepatic toxicity than the mean TAD alone [18]. In other words, it is feasible to escalate the tumour dose while maintaining acceptable non-tumoral liver exposure in selected cases. However, indiscriminate dose escalation risks hepatic decompensation.

A strong correlation (*r* = .857) was observed between pre-treatment MAA-based and post-treatment Y-90 PET-based TAD estimates. This level of agreement is not inherently expected, given the known physical and methodological differences between MAA particles and Y-90 glass microspheres, including differences in particle size distribution, particle number, and embolic characteristics, as well as the use of different imaging modalities (SPECT/CT versus PET/CT). Several factors may explain this finding. First, both MAA particles and Y-90 microspheres are primarily distributed according to hepatic arterial perfusion. In the absence of significant hemodynamic changes between simulation and treatment, macrovascular flow patterns remain the dominant determinant of intrahepatic distribution. Second, strict reproducibility of catheter positioning and injection technique between the planning and treatment procedures likely contributed to the observed concordance. Third, the relatively low embolic load of glass microspheres minimizes flow alteration during administration, thereby preserving the predictive value of the MAA distribution. Finally, quantitative hybrid imaging techniques (SPECT/CT and PET/CT) improve spatial and dosimetric accuracy compared to earlier non-quantitative approaches. Nevertheless, it should be emphasised that correlation does not imply perfect agreement and that systematic differences between MAA and Y-90 distributions may still exist at the level of individual lesions.

There were several limitations to the current study. The major drawbacks were the retrospective design, moderate sample size, relatively high prevalence of extrahepatic disease, and the use of mean/median TAD without routine voxel-level analysis of intratumoral heterogeneity. The high prevalence of extrahepatic disease and advanced liver tumor burden in this cohort reduces the statistical power to measure the intrahepatic treatment effects. This may mask the potential benefits of a higher TAD among patients with a more liver-dominant disease. Furthermore, the group sizes (TAD < 189 Gy vs. ≥189 Gy) were unequal, which could affect the estimates of the effect size and widen the confidence intervals. Due to the retrospective nature of the study and the absence of routine voxel-level quantification, the lesion-level microsphere distribution and size-based compartmental analysis could not be performed. Future prospective studies integrating voxel-based PET dosimetry may better elucidate the relationship between microsphere number, lesion size, and response. Although a strong correlation was observed between pre-treatment MAA-based and post-treatment PET-based TAD estimates, this does not imply complete quantitative agreement. Differences in particle characteristics, microvascular distribution and imaging modalities may introduce systematic biases, particularly at the lesion level. These biases may not be fully captured by correlation analysis alone. Systematic differences between methods may lead to reclassification of individual patients around a given dose threshold. However, the use of planning-based dosimetry in the present study reflects real-world clinical decision-making, and the findings should be interpreted as an evaluation of the clinical utility of a guideline-referenced threshold rather than a definitive biological dose–response relationship. Another factor that may influence microsphere distribution is the use of different vial calibrations. In the present study, both week 1 and week 2 TheraSphere vials were used. Week 2 vials contain a higher number of microspheres per unit activity, which may increase embolic load and alter microvascular distribution compared to week 1 vials. This difference has been reported to potentially affect the concordance between MAA-based simulation and Y-90 microsphere deposition. The use of mixed vial types may have introduced additional variability, particularly at the lesion level, which could not be fully accounted for in this retrospective analysis. Another potential confounding factor is prior exposure to bevacizumab. Prior bevacizumab exposure may reduce tumor vascularity and alter arterial perfusion, potentially affecting both MAA distribution and Y-90 microsphere targeting. Due to heterogeneity in treatment timing, this effect could not be systematically evaluated and represents a potential confounder.

An important methodological consideration in the present study is how to handle multifocal disease. Although TAD was initially calculated at the lesion level, patient-level analyses were performed using the median TAD across all lesions, with no specific correction for underdosed tumors. This approach may obscure clinically relevant dose heterogeneity within patients, particularly in cases where a subset of lesions receives suboptimal radiation. Consequently, potential dose–response relationships may be attenuated and the impact of insufficiently treated lesions may not be fully captured in survival analyses. Future studies that incorporate lesion-level or voxel-based dosimetry metrics, such as minimum TAD or dose–volume parameters, could provide a more accurate evaluation of the relationship between radiation dose distribution and clinical outcomes. Finally, differences in microsphere type (glass vs. resin), dosimetry technique, imaging response criteria and patient population make comparisons across studies complicated. Nevertheless, real-world data differs from that of trials, which only include ideal patients. To define optimal dosing strategies, future work is needed that uses voxel-level dosimetry, standardised response metrics and integrated systemic-local treatment approaches.

In conclusion, in this heavily pretreated population with advanced, extrahepatic disease, escalation of TAD beyond 189 Gy did not translate into a survival benefit, underscoring the dominant role of systemic disease burden over locoregional dose intensification.

## Data Availability

The datasets used or analyzed in the current study are available from the corresponding author on reasonable request.
